# Task-Level Strategies for Human Sagittal-Plane Running Maneuvers Are Consistent with Robotic Control Policies

**DOI:** 10.1371/journal.pone.0051888

**Published:** 2012-12-20

**Authors:** Mu Qiao, Devin L. Jindrich

**Affiliations:** 1 Kinesiology Program, School of Nutrition and Health Promotion, Arizona State University, Tempe, Arizona, United States of America; 2 Department of Kinesiology, California State University San Marcos, San Marcos, California, United States of America; Universidad Europea de Madrid, Spain

## Abstract

The strategies that humans use to control unsteady locomotion are not well understood. A “spring-mass” template comprised of a point mass bouncing on a sprung leg can approximate both center of mass movements and ground reaction forces during running in humans and other animals. Legged robots that operate as bouncing, “spring-mass” systems can maintain stable motion using relatively simple, distributed feedback rules. We tested whether the changes to sagittal-plane movements during five running tasks involving active changes to running height, speed, and orientation were consistent with the rules used by bouncing robots to maintain stability. Changes to running height were associated with changes to leg force but not stance duration. To change speed, humans primarily used a “pogo stick” strategy, where speed changes were associated with adjustments to fore-aft foot placement, and not a “unicycle” strategy involving systematic changes to stance leg hip moment. However, hip moments were related to changes to body orientation and angular speed. Hip moments could be described with first order proportional-derivative relationship to trunk pitch. Overall, the task-level strategies used for body control in humans were consistent with the strategies employed by bouncing robots. Identification of these behavioral strategies could lead to a better understanding of the sensorimotor mechanisms that allow for effective unsteady locomotion.

## Introduction

How do humans control walking and running? Although locomotion is an important motor behavior, the strategies used for body control are not well understood. Legs can act like springs to store and release mechanical energy and reduce metabolic cost [1]. Spring-like properties can describe leg mechanics, and may potentially be control targets [2]. For example, humans adjust effective leg spring stiffness to maintain a relatively constant center of mass (COM) displacement [3]. Although legs may often resemble symmetrical, Hookean springs, asymmetries between compression and thrust mechanics may also be functional [4].

In addition to force, energy, and power requirements, constant-speed locomotion also requires stability. Stability can be defined as maintaining non-divergent patterns of cyclic movement over time [5]. Challenges to stability, as posed by age or disease, are associated with shorter strides and wider stance [6], [7]. However, the rules governing how leg spring stiffness, foot placement, ground reaction forces (GRFs), muscle activity and other variables are adjusted to stabilize locomotion and maneuver remain largely uncharacterized [8].

A spring-mass model, where the body is approximated as a point mass and the leg approximated by a linear spring, can successfully describe many aspects of locomotion mechanics using a small number of parameters [9], [10]. Spring-mass systems like runners can benefit from passive mechanical stability, augmented by relatively simple adjustments like leg retraction and changes to end-of-swing leg stiffness [11], [12]. However, passive dynamic stability may be limited to specific ranges of parameters, and only be able to reject small perturbations to movement [13]–[16]. Consequently, anticipation and feedback are also necessary for robust bipedalism in a variable environment [17].

Although anticipation is often used for maneuvers, robotics research has shown that independent, distributed feedback rules can stabilize bipedal locomotion. For example, Raibert and colleagues built monopods that bounced on leg springs similar to animal running and were stabilized by independently controlling hopping height with leg thrust force, forward speed with foot placement, and body attitude with hip moments [18]. For example, to accelerate/decelerate the monopod places its foot behind/in front of a reference point, and the more a foot is behind/in front of the reference point the more speed is increased/decreased during stance. These rules can be considered “task-level” strategies because they focus on the task of stabilizing the COM, but could be achieved in different ways (e.g. using a linear actuators in telescoping legs or rotational actuators in jointed legs). Analogous principles can be used for bipedal robot walking, where leg placement within a defined capture region can be used to resist perturbations [19]. Walking humans also adjust foot placement relative to an estimated position to maintain balance in the sagittal plane [20]. That feedback strategies can stabilize both walking and running robots presents the question of whether humans could employ similar mechanisms during running.

This study sought to describe the strategies used by humans for body control during running. Specifically, we tested the hypothesis that humans actively change running height (COM apex during flight), forward speed, and body attitude using strategies consistent with those used by Raibert’s robots. We chose these specific control laws because they are simple enough to serve as experimentally testable hypotheses, have rules to account for both translational and rotational stability, and, importantly, have been demonstrated to work together to stabilize bouncing robots over level ground. They therefore represent strategies that, together, are sufficient to provide a basis for enhancing the stability of spring-mass systems. In addition, they are relationships that could potentially be available to organize more complex maneuvers. We therefore investigated whether (1) running height is related to leg thrust, and not the alternative strategy of changing stance duration [14]. In addition, we sought to determine whether legs act as symmetrical springs during maneuvers, or whether leg properties differ during the compression and thrust phases of stance. We also determined whether (2) speed changes are linearly correlated to foot placement, i.e. the distance from the center of pressure (COP) to a “Neutral Point” (NP). Finally, we tested whether (3) a proportional-derivative (PD) function can describe the relationship between hip moments and trunk pitch during running tasks involving changes to running height, speed, and pitch.

## Materials and Methods

### Tasks and Participants

**Figure 1:**
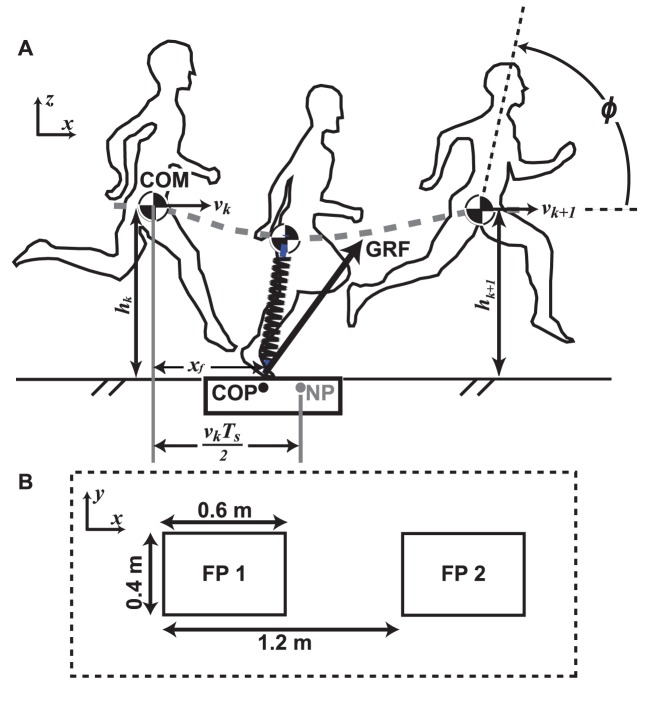
Methods used to evaluate body control strategies for running. (A) Definition of parameters: running height (*h_k_*), COM horizontal speed (*v_k_*), virtual leg (spring connecting COP and COM), leg force (projection of GRF onto the virtual leg), components of the NP strategy for maintaining forward running speed, and definition of body orientation (ϕ). (B) Experimental coordinates and force platform setup. Dashed line box indicates dimensions of rubber mat used to obscure the force platforms.

We recruited 16 males (age 27±4 years; body mass (bm) 70±8 kg; height (bh) 177±7 cm, leg length (*l*) 96±7 cm, from the sole of the foot to the greater trochanter, mean±std) during five tasks requiring changes to COM height and running speed, while maintaining body orientation: constant-average-speed running (CSR), stepping up (SU), stepping down (SD), acceleration (ACC), and deceleration (DEC). Participants selected a comfortable speed during approximately 10 warm-up trials. For the SU and SD tasks, a wooden runway (20 cm high) was placed either 94 cm behind the 2^nd^ platform (SU) or in front of the 1^st^ platform (SD; Fig. 1). For SU only the step before takeoff (TO), i.e. before the change in ground level, and for SD only the step after landing was analyzed. For ACC, CSR, and DEC both steps were used for analysis. For ACC and DEC, participants accelerated (decelerated) to their comfortable speed between two ground markers 5 meters apart. The task order was randomized, and a short (5 min) rest was provided if requested. Because participants could not see the locations of force platforms under the rubber mat, we were able to select starting positions that maximized the probability of successive steps on both force platforms (FPs) without informing the participants about the locations of the FPs or the purpose of the adjustments to starting position. Each participant performed approximately 50 trials to result in 25 successful trials: 5 replicates for each task. Successful trials were defined as trials where one foot stepped within the area of the first FP and the other foot stepped within the second FP. All procedures were approved by the Arizona State University Institutional Review Board.

### Data Collection

We collected whole-body 3-D kinematics by tracking 39 markers (Plug-In-Gait marker set) from a 10-camera motion tracking system at 120 Hz (VICON® 612, Oxford Metrics Ltd., Oxford, UK). For all tasks, participants ran from a starting position, over two 0.6×0.4 m FPs sampling at 3000 Hz (FP4060-NC, Bertec Corporation, Columbus, OH, USA) covered by 120×160 cm, 2 mm-thick rubber mat (Ironcompany, Lafayette, CA, USA) to a stopping position approximately 10 m away (Fig. 1AB). Dynamic tests using forces approximating human running peak forces (1900N) showed that the mats caused negligible force attenuation (<0.5%) and cross-talk (<0.6%).

### Data Analysis

The COM was calculated by segmental averaging after inverse kinematics. Its trajectory was then refined by taking GRF into account, allowing robust calculation of velocity at touch down (TD) and TO [21]. Kinetic and kinematic data were filtered using a 4^th^-order low-pass zero-lag Butterworth digital filter at 60 Hz and 33 Hz, respectively. Inverse dynamics was used to calculate net joint moments [22]. Positive hip, knee, and ankle moments indicated extension, flexion, and plantar flexion, respectively.

Data analysis was performed in MATLAB (R2012a, Mathworks, Natick, MA, USA). Statistical power (0.93 across all participants by assuming a low effect size of 0.15) for the regression analysis was calculated by G*Power (3.0.10, Franz Faul, University Kiel, Germany).

**Figure 2:**
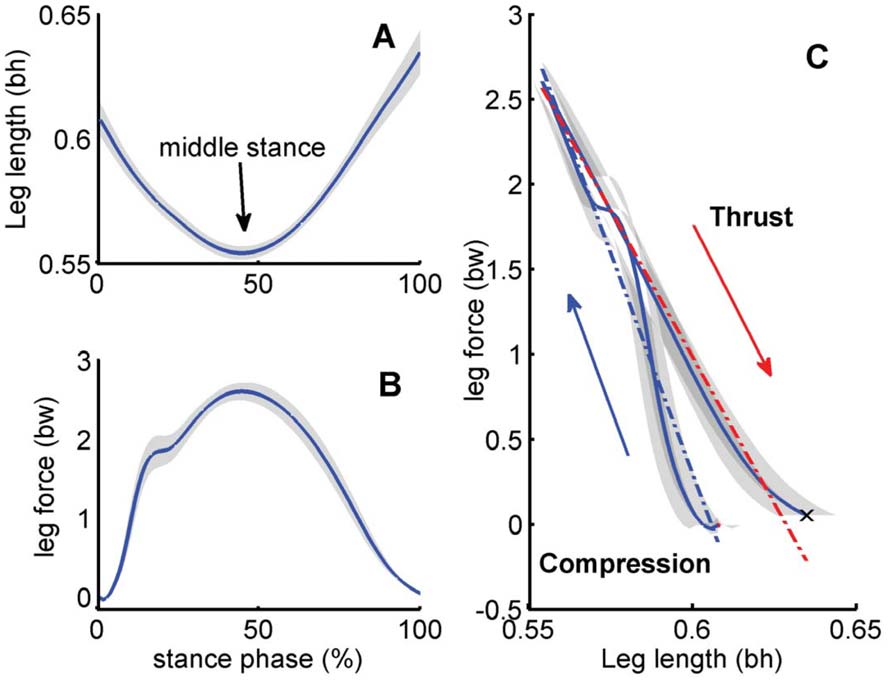
Profiles of virtual leg length, leg force, and work loops during stance phase. (A) Virtual leg length during stance. Mid-stance was defined as the instant of minimum leg length. (B) Leg force during stance. (C) The relationship between leg length and force. In the compression (loading) phase, leg length decreased and leg force increased. In the thrust (unloading) phase, leg length increased and leg force decreased. A linear fit was used to describe the relationship between leg length and force in each phase (blue in loading, red in unloading). The slope of that linear fit was defined as the leg spring stiffness. All trials within each condition were averaged first, and then averaged across all conditions and participants. Shaded areas indicate mean±s.e.m.

To evaluate the running height hypothesis (1), we used repeated-measures ANOVA to compare leg force, stiffness and stance duration during CSR, SU, and SD [23]. Post-hoc comparisons were based on a Bonferroni procedure with Šidák correction (*p*<(1*–*(1*–*α)*^1/c^*), c = (a*–*1)·a/2, a is the number of levels). Running height was calculated by fitting a 2^nd^ order polynomial to flight-phase vertical COM position and selecting the maximum. Leg force was calculated as the projection of the GRF onto a “virtual leg,” a vector connecting the COP to COM (Fig. 1A). The beginning of stance, TD, was identified as when the vertical GRF increased for 60 ms, and mid-stance as the instant of minimum “virtual leg” length. The period from stance onset, TD, to mid-stance when the leg length was minimum was defined as “compression;” the period from mid-stance to TO as “thrust” (Fig. 2A). The average leg force in compression and thrust phases were defined as compression and thrust forces, respectively (Fig. 2B). We calculated leg stiffness by linear regression of leg force relative to virtual leg length during thrust and compression (Fig. 2C).

To evaluate the running speed hypothesis (2), we calculated forward speed and foot placement during CSR, ACC and DEC. Raibert’s control of speed takes the form.
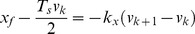
(1)where *x_f_* is average COP position over stance relative to the COM, *k_x_* is a gain, *v_k_* and *v_k+_*_1_ are COM horizontal speeds in flight in two consecutive steps. The NP relative to the COM was calculated as half the product of *v_k_* and the subsequent stance duration (*T_s_*; Fig. 1A). When COP and NP coincide, there is zero net acceleration. We used linear regression to test the relationship between *x_f_ –T_s_·v_k_/2* and *v_k+1_*–*v_k_*.

To evaluate the body orientation hypothesis (3), body pitch angle, ϕ, was estimated with a vector between the midpoints of the four pelvic markers and the midpoint of the 7^th^ cervical vertebra and clavicular markers (Fig. 1A). Raibert’s control of body orientation takes the PD form.
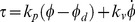
(2)where *τ* is the hip reaction moment to the upper torso, equal to the sum of both hip joints’ extension moments. Positive τ was defined as causing positive changes to ϕ. Negative *k_p_* and *k_v_* therefore suggests error attenuation, analogous to negative feedback. ϕ_d_ is the desired pitch angle, and *k_p_* and *k_v_* are gains (Fig. 1A). We tested the relationship between *τ*(*t*), ϕ(*t*), and its time derivative, 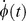, for Eq. 2 by using linear regressions for each trial during stance. We also used *t*-tests to examine whether the *k_p_* and *k_p_* were significantly different from zero.

To account for size differences, we normalized force by body weight (bw, bm·*g*), apex height by body height (bh), and expressed speed as a Froude number (Fr), calculated as 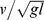, where *l* is leg length [24]. All values are mean±std except where indicated.

## Results

### Running Height

**Figure 3:**
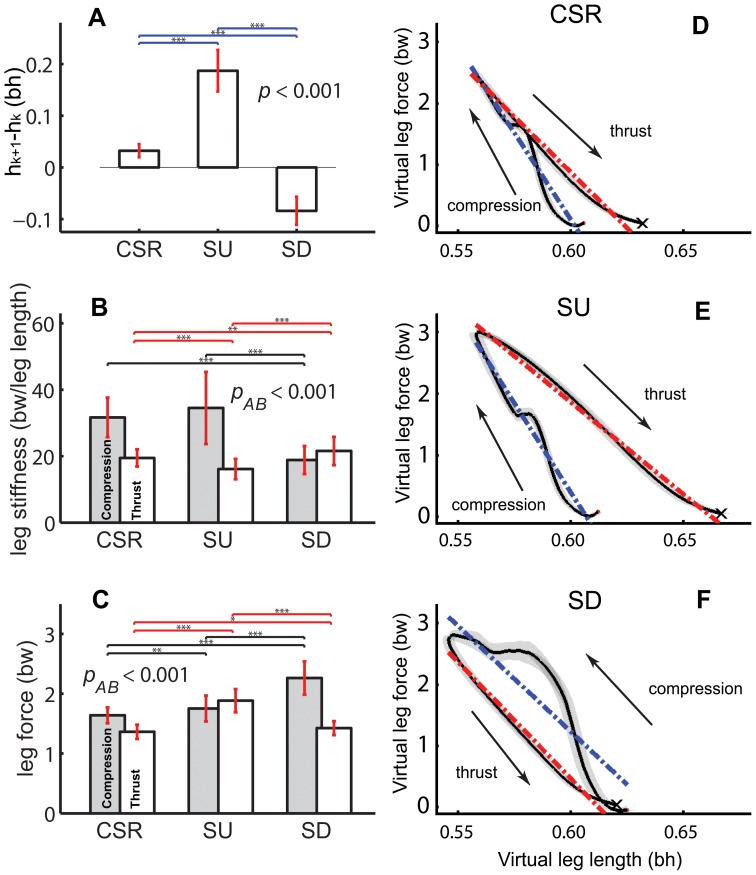
Variables determining COM apex. (A) COM height change in two consecutive flight phases (*h_k+1_– h_k_*; Fig. 1A). Changes to leg stiffness(B) and force(C) during the compression and thrust phases of stance for CSR, SU and SD. The relationship between leg force and virtual leg length during stance in CSR (D), SU (E) and SD (F). Interaction effect *p_AB_* was obtained by factorial repeated ANOVA (task and phase). * means *p*<0.05, ** means *p*<0.01, and *** means *p*<0.001.

Changes to COM running height were associated with changes to leg force and not the alternative of changing stance period. SU and SD involved significant changes of COM height in two consecutive flight phases (*h*_k+1_–*h*_k_; *p*<0.001; Fig. 3A). Stance periods during SU (250±41 ms) were not significantly different from CSR (260±28 ms, paired *t*-test, *p* = 0.06). Stance periods during SD dropped by 7% to 243±34 ms with respect to CSR (*p*<0.01). For all tasks, on average, the instant of mid-stance was significantly later than the instant of peak leg force (5±4% stance duration, paired *t*-test, *p*<0.001). For individual tasks, mid-stance preceded peak force by 8±6% (ACC), 0±3% (CSR), −12±12% (DEC), 3±8% (SU), −23±9% (SD) stance duration. Duty factors were not significantly different among running height tasks (*p* = 0.16; 0.38±0.04, 0.39±0.03, and 0.40±0.03 in ACC, SU, and SD, respectively, mean±std). Overall, these data support Hypothesis 1 that force magnitude, not duration, is used to change running height.

Humans did not alter leg force uniformly over stance, but appeared to independently change leg behavior during compression and thrust phases. Under most conditions, a linear relationship between leg force and length in loading and unloading could be effectively described as a “stiffness” (Fig. 3D–F). However, different tasks involved alterations to leg stiffness during separate phases of stance. SU involved significant changes to leg stiffness relative to CSR during thrust (17±8% decrease, Fig. 3B, *p*<0.001), without significant changes during compression (9±30% decrease, Fig. 3B, *p* = 0.26). This resulted in increases in average thrust forces that were 5-fold larger (39±13%) than increases in compression (7±8%, Fig. 3C, *p*<0.001). In SD, compression leg stiffness showed an abrupt decrease in stiffness near mid-stance, causing overall leg stiffness during compression to decrease significantly (41±8%, Fig. 3B, *p*<0.001), and average compression force to increase 39±13% relative to CSR (Fig. 3C, *p*<0.001). In contrast, changes to leg stiffness during thrust were small (9±13% increase, Fig. 3B, *p*<0.05). Consequently, SU and SD maneuvers both involved increases in leg forces accompanied by decreases to leg stiffness, but during different phases of stance.

### Speed

**Figure 4:**
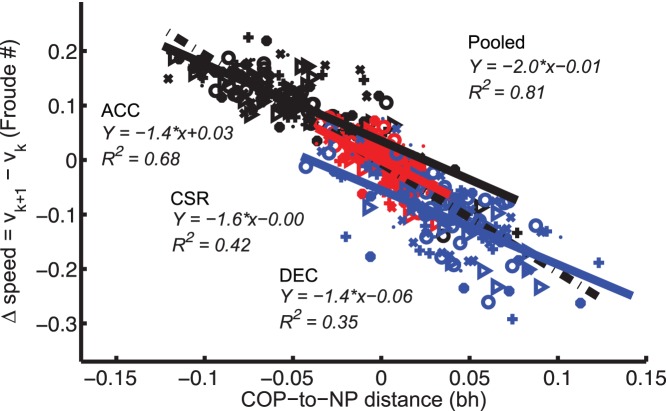
Foot placement changes associated with speed change. Relationship between COP-to-NP distance and speed increment (*v_k+1_– v_k_*) for ACC, DEC and CSR. For scatter plots, each participant is represented by a different symbol and tasks by colors (Black: ACC; red: CSR; blue: DEC).

Foot placement used for ACC, CSR, and DEC was consistent with a NP strategy (supporting Hypothesis 2). Linear regressions performed within individuals on COP-to-NP distance and speed increment were all significant (slope of −2.1±0.25Fr/bh, *R* ^2^ = 0.81±0.16, *p*<0.001), as was a linear regression using pooled data (Fig. 4). Moreover, regressions within tasks yielded significant correlations between speed increment and COP-to-NP distance (slope of CSR −1.6Fr/bh, *R* ^2^ = 0.42; ACC −1.4Fr/bh, *R* ^2^ = 0.68; DEC −1.36Fr/bh, *R* ^2^ = 0.35, *p*<0.001).

**Figure 5:**
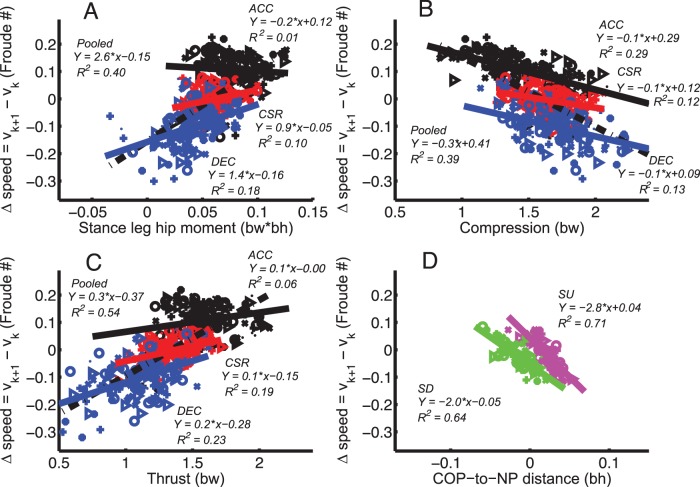
Variables associated with speed increment. Relationships between speed increment (*v_k+1_– v_k_*) and independent variables (A) stance leg hip moment, the time average of hip flexion (+)/extension (*–*) moment during stance phase, (B) compression, (C) thrust force for ACC, DEC and CSR. (D) Relationship between COP-to-NP distance and *v_k+1_– v_k_* for SU (magenta) and SD (green).

Anticipated maneuvers involved simultaneous changes to more than one parameter. For example, stance leg hip moment, which was the time average of hip moment during stance phase and could contribute to acceleration, was significantly correlated to speed increment (Fig. 5A, *p*<0.001). However, regressions within tasks showed significant but weak relationships (*R^2^* = 0.01, *p*<0.001 in ACC, *R^2^* = 0.10, *p*<0.001 in CSR, *R^2^* = 0.18, *p*<0.001 in DEC), suggesting that between-task differences reflected task-, not speed-dependent changes to hip moment. When correlating time-averaged ankle moment during the stance phase with speed increment, the same pattern as hip moment was observed: the pooled regression had a slope of 2.9Fr/(bw·bh), *R* ^2^ = 0.37, but within-task fits were poor (1.1Fr/(bw·bh), *R* ^2^ = 0.13 ACC; 0.46Fr/(bw·bh), *R* ^2^ = 0.03 CSR; 1.5Fr/(bw·bh), *R* ^2^ = 0.09 DEC). Similarly, although ACC and DEC involved changes to leg force relative to CSR (16±13% increase in thrust, *p*<0.001, and 13±12% decrease, *p*<0.001, in compression during ACC, 16±7% decrease in thrust, *p*<0.001, and 4±8% increase in compression, *p* = 0.05, during DEC), leg forces were only weakly correlated to speed increment within each condition (*R* ^2^ of 0.29, 0.12, 0.13 for compression and 0.06, 0.19, 0.23 for thrust for ACC, CSR, and DEC, respectively; Fig. 5BC).

SU and SD tasks were also associated with foot placement changes (0.02±0.016 bh anterior and 0.014±0.017 bh posterior to NP for SU and SD, respectively). However, these shifts did not substantially alter the relationship between speed increment and COP-to-NP distance from CSR, ACC, and DEC, apart from a steeper slope for SU (compare Fig. 5D with Fig. 4).

### Body Orientation

**Figure 6:**
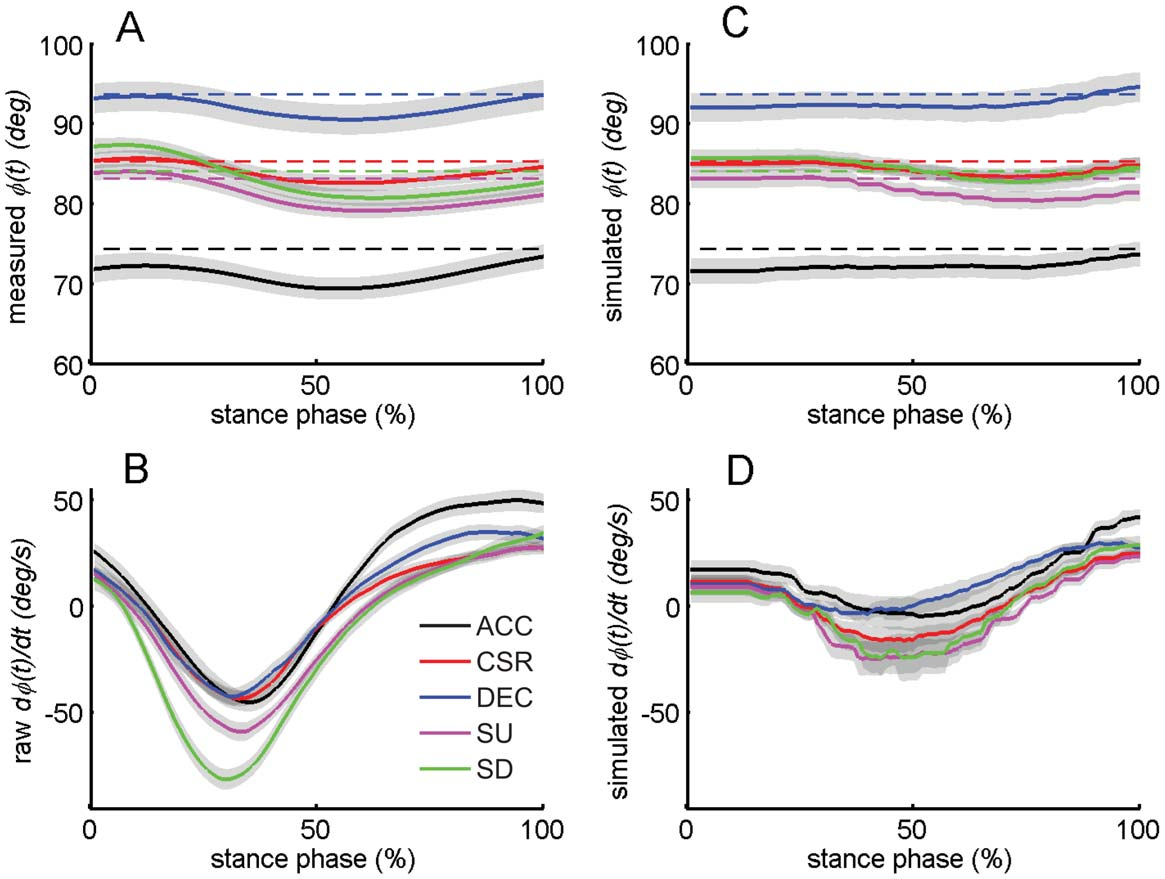
Relationship of hip moments to body orientation. Measured body pitch angle ϕ(*t*) (A), and angular speed 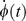 (B) during stance. Line colors corresponded to the tasks (Black: ACC; red: CSR; blue: DEC; magenta: SU; green SD). For ϕ(*t*) the reference angles ϕ_d_ from each task are indicated by the dashed horizontal lines. Pitch angle ϕ(*t*) (C) and angular speed 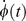 (D) predicted by Eq. 2 in stance. Each trial was simulated from the initial conditions of ϕ and 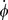 at TD. For each participant the simulation was implemented under ode45 function of MATLAB 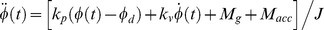. *J* is the pitch moment of inertia of the upper extremities relative to the hip in the sagittal plane. *M_g_* is the moment relative to hip caused by the upper body’s COM, and *M_acc_* is the moment relative to the hip caused by the acceleration of upper body’s COM in the sagittal plane. For each participant all trials within each task were averaged, then averaged across all participants within a task. Shaded areas indicate mean±s.e.m.

**Figure 7:**
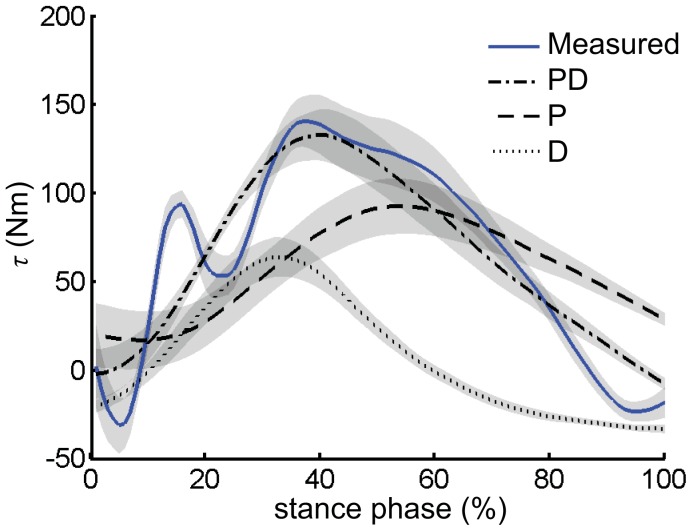
Comparison between measured hip moments and predicted hip moments. Measured hip moment was calculated as the sum of both legs’ hip flexion/extension moments (blue solid). τ (PD, dash and dotted) was predicted by (Eq. 2). P (dashed) is the proportional portion of τ, while D (dotted) is the derivative component of τ. Measured hip moment, τ, P, and D were first averaged from all trials within each task for each participant, and then averaged across all participants and tasks. Shaded areas are mean±s.e.m.

Maintenance of body pitch was consistent with a PD feedback rule to determine hip moments. During stance, ϕ(*t*) decreased (i.e. forward lean) until approximately mid-stance before increasing again (Fig. 6AB). The shift from negative to positive rotational velocity was caused by positive hip extension moments (Fig. 7). Gains in Eq. 2 fit to each trial in stance were negative (*k_p_* = −16.4±14.5 N·m/deg, *t*-test, *p*<0.001, *k_v_* = -1.2±0.33 N·m·s/deg, *p*<0.001, *R* ^2^ = 0.51±0.14 across all tasks, Table 1 for individual task), suggesting that hip moments acted to resist deviations from the reference angle.

**Table 1:** Parameters in Eq. 2 for different tasks.

tasks	*k_p_* (N·m/deg)	*k_v_* (N·m·s/deg)	*φ_d_* (deg)	*R* ^2^
ACC	−25±17	−1.3±0.73	74±6.7	0.67±0.17
CSR	−20±17	−1.3±0.45	85±4.6	0.57±0.19
DEC	−10±18	−0.8±0.38	94±7.1	0.42±0.16
SU	−21±18	−1.3±0.35	83±5.1	0.65±0.21
SD	−14±15	−1.2±0.84	84±5.3	0.40±0.18

The hypothesis that the relationship between hip moments and trunk movement is consistent with a PD rule was supported by the failure of other relationships to yield better fits. Both P-only (*k_p_* = −14.1±12.4 N·m/deg, *p*<0.001, *R* ^2^ = 0.15±0.14) or D-only (*k_v_* = −1.14±0.4 N·m·s/deg, *p*<0.001, *R* ^2^ = 0.28±0.09) relationships resulted in significantly poorer fits compared to PD. Moreover, a PID (I, integrative) relationship did not improve fits significantly (*R* ^2^ = 0.64±0.13, *p* = 0.06, *F*-test on higher order coefficient, *k_p_* = −30±15 N·m/deg, *p*<0.001, *k_v_* = −1.0±0.5 N·m·s/deg, *p*<0.001). The integrative parameter was also not significantly different from zero (*k_i_* = −1.9±3.1 N·m/(deg·s), *p* = 0.05, *t*-test).

## Discussion

Although anticipated maneuvers involved task-dependent changes to multiple parameters, the underlying relationships among parameters remained consistent with the strategies used by Raibert’s robots. Changes to COM height require changes to vertical force impulses [17]. For both SU and SD tasks, humans changed leg force magnitude, not duration, duty factor or peak phase, to change running height. Speed changes were correlated with foot placement but not strongly with stance leg hip moment or leg force. A substantial component of hip moments could be described by a PD relationship to body pitch.

### Running Height

Increases in thrust force during SU or compression during SD were both associated with decreased leg stiffness that facilitated energy release or absorption (Fig. 3). For example, increasing thrust force in SU resulted from more leg compliance and greater excursion during thrust than during compression, forming a clockwise work loop between leg force and virtual leg length and net work production, indicated by the area within the work loop. For SD the behavior of the leg during compression showed a shift near mid-stance to a period of high compliance and negative work resulting from a counter-clockwise work loop [25]. Negative work during compression was similar to birds stepping down, where energy was absorbed in the hip [26].

Although the stance leg is often described with a single stiffness [3], these patterns during maneuvers appear to represent functionally relevant asymmetries between compression and thrust [4]. During SU overall stance stiffness was not different from CSR, consistent with the unchanged stiffness found for smaller (10 cm) steps [17]. However, apparently independent changes to stiffness during thrust and compression for SU and SD, respectively, suggest that overall stiffness may not sufficiently characterize leg mechanics during maneuvers.

### Speed

Humans changed speed during ACC or DEC by adjusting foot placement consistent with a “Neutral Point” strategy (Fig. 4). Primarily using foot placement to change speed could allow human legs to function like the telescoping legs of Raibert’s robots. For example, during long jumps a lower angle of attack (AOA; i.e. positive “COP-to-NP distance”) results in lower horizontal velocity and decreased jumping distance [27]. Foot placement can be used to determine the conversion between potential and kinetic energy [21], [28]. However, other mechanisms such as increasing leg length in thrust are also available for controlling energy [16]. We observed strategies consistent with a linear relationship and the legs acting as telescoping springs. The observed changes in foot placement are consistent with those found during walking, where the COM acceleration is proportional to the horizontal distance between COP and COM [29]. Foot placement during walking is also used to maintain lateral stability, potentially reflecting relatively simple predictive rules [30]. Changes to foot placement can be effective for stabilization because initial position can affect subsequent joint mechanics during stance [31]. Moreover, horizontal plane maneuvers are also associated with changes to foot placement, although the factors that determine foot placement selection have not been determined [32]. This presents the possibility that shared strategies are used for body control in many contexts.

A telescoping spring-mass, or “pogo stick,” strategy involves generating different GRFs by changing leg placement and using axial leg forces. Using a “pogo stick” strategy could be beneficial because it decouples torso translation from rotation and could facilitate the use of independent strategies for both aspects of movement. The alternative strategy could be thought of as analogous to a “unicycle,” where moments generated by the crank are translated into GRFs by the wheel, but the point of contact of the wheel progresses along the ground, which is in contrast with the “pogo stick” whose contact point remains constant. However, behavior similar to a “unicycle” strategy was less evident. Fore-aft speed increments were not strongly associated with stance leg hip moment without alterations to foot placement (Fig. 5A). This does not, however, diminish the role of joint moments for powering running and compensating for changes such as inclines [33]. Hip flexors contribute to braking in early stance, and plantar flexors to COM propulsion in the second half of stance [34]. Contributions of joint moments to running were also not limited to the hip. For the distal joints in our ACC task, ankle moments accounted for 37% of the speed increment, potentially preventing running height from decreasing.

Although overall leg mechanics may be organized to achieve specific task goals, individual joints may function differently during unsteady locomotion. Proximal joints may be used in a more feed-forward manner than distal joints, where load sensitivity suggests feedback contributions [8], [26]. Effective feed-forward strategies may be important for successful human maneuvers because time delays could limit the effectiveness of neural compensations [35]. Appropriate anticipatory strategies may also be important for preventing tissue overloading and injuries during maneuvers [36].

Although anticipated maneuvers were associated with task-related changes to leg force and hip moment, these changes did not appear to affect the relationship of speed increment to foot placement (Fig. 5). Task-specific offsets to locomotion parameters may therefore be superimposed onto body control strategies, potentially representing a de-coupling of task-specific changes from underlying locomotory patterns [37].

### Body Orientation

The relationship between body pitch and hip moments was consistent with a negative feedback PD strategy common to many robotic applications, as evidenced by the ability of a PD relationship to generate reasonable hip moments (Fig. 7). To further test the potential of a simple PD relationship to regulate body pitch angle, we simulated the trunk motion starting at its angle and angular velocity at the beginning of stance, and integrated forward by using net pitching moment, calculated as the sum of 1) τ determined from Eq. 2, 2) gravitational moment caused by the upper body’s COM relative to the hip in the sagittal plane, and 3) inertial moment due to the acceleration of upper torso. The resulting angle and angular velocity profiles demonstrated that the pattern of motion was reasonable: simulated body pitch movement showed similar features to measured body pitch. The action of a PD feedback relationship could lead to a re-entrant pattern of trunk rotational position/velocity: conditions at TO facilitate appropriate initial conditions for TD of the following step (Fig. 6CD).

Mechanical factors such as segmental inertias and intrinsic musculoskeletal properties can contribute to stability at very rapid timescales, and could explain the ability of PD relationship without a time lag to partially describe hip moments during stance [38], [39]. However, behavior consistent with PD control can also be found in many other contexts involving neural-mediated compensations. Flies use behavior consistent with PD control with delays of several wing beats to overcome yaw perturbations and maintain their original movement direction and orientation [40]. Cockroaches also exhibit behavior consistent with PD control during wall following [41], [42]. Humans can use relatively rapid, pre-programmed reactions to compensate for perturbations [43], [44]. However, the simple PD relationship we tested was not fully able to predict hip moments and trunk motions (Fig. 6CD). The high frequency oscillation of hip moments at the beginning of the stance phase was caused by the initial peak of the vertical GRF associated with the heel to toe contact of foot to the ground (Fig. 7). That moment cannot be fully accounted for by the PD controller from body pitch kinematics because there is no such a rapid oscillation in body pitch angle. Consequently, a fuller description of body orientation may require the inclusion of higher-order feedback pathways that account for known time delays associated with neural processing.

### Potential Implications

Our experiments studied body control during anticipated changes to running height or speed. However, we observed that humans changed parameters that were consistent with those used by the distributed, independent feedback rules used by Raibert’s robots to maintain stability. This presents the possibility that locomotion in humans involves task-level strategies that relate desired changes in movement trajectories to behavioral adjustments. The ability of task-level strategies to control locomotion, a mechanically complex behavior involving many muscles, could be facilitated by physiological organization at several levels. Muscle and reflex properties can contribute to mechanical stability of the limbs [45]. Muscle groups may be activated to achieve specific movement objectives [46]. Synergistic activity at the spinal or brainstem level could also contribute to the sensing and control of higher-order parameters such as leg orientation and endpoint [47]. Consequently, task-level policies could be separated from the complexity of neuromuscular structure and dynamics.

Characterizing the kinematic and dynamic mechanisms used by humans to maneuver and maintain stability could help to better understand how sensory information is interpreted and processed to achieve effective motor output [48]. However, our correlational study of sagittal-plane maneuvering behavior was not designed to provide a test of the underlying control mechanisms used by humans to maintain stability or execute maneuvers. Although our results are consistent with distributed feedback rules, they do not exclude other motor control structures, such as central, model-based control, that could also result in the observed correlations. Moreover, our results also do not exclude the possibility that locomotion control is task-specific, phase-dependent, or involves several mechanisms operating hierarchically or in parallel. Our experiments on anticipated maneuvers also do not establish that simple control rules are sufficient for, or employed by, humans to maintain stability. Perturbation and neurophysiological studies will be necessary to address these limitations and distinguish among the many potential mechanisms that could underlie unsteady locomotion performance.

Our results suggest that humans show body control strategies that result in relationships among movement parameters that are consistent with the distributed feedback rules used by Raibert’s robots. Moreover, the results revealed that compression and thrust phases of stance may be independently modulated, and revealed some of the task-based changes to parameters such as hip moment and foot placement associated with maneuvers. However, the possibility of task-level rules does not diminish the importance of anticipation for stability and maneuverability. For example, maintaining speed through foot placement relative to NP requires estimation of NP. In complex natural environments, locomotion must be continuously adjusted to account for changes due to substratum compliance and incline [3], [33]. Anticipatory, or proactive, adjustments and learning provide continuous modulation of motor output [49]. Whether running humans use reactive strategies in response to external perturbations similar to the proactive strategies we studied remains to be determined.
